# Neurofilament light as a blood biomarker for neurodegeneration in Down syndrome

**DOI:** 10.1186/s13195-018-0367-x

**Published:** 2018-04-10

**Authors:** Andre Strydom, Amanda Heslegrave, Carla M. Startin, Kin Y. Mok, John Hardy, Jurgen Groet, Dean Nizetic, Henrik Zetterberg, The LonDownS Consortium, Andre Strydom, Elizabeth Fisher, Dean Nizetic, John Hardy, Victor Tybulewicz, Annette Karmiloff-Smith, Sarah Hamburg, Rosalyn Hithersay

**Affiliations:** 1 Department of Forensic and Neurodevelopmental Sciences, Institute of Psychiatry, Psychology and Neuroscience, King’s College London, 16 De Crespigny Park, London, SE5 8AF UK; 2 Division of Psychiatry, University College London, London, UK; 3 The LonDownS Consortium, Department of Forensic and Neurodevelopmental Sciences, Institute of Psychiatry, Psychology and Neuroscience, King’s College London, Denmark Hill, London, SE5 8AF UK; 4 Department of Molecular Neuroscience, Institute of Neurology, University College London, London, UK; 5 Division of Life Science, Hong Kong University of Science and Technology, Hong Kong, Hong Kong, Special Administrative Region of China; 6 Reta Lila Weston Institute, Institute of Neurology, University College London, London, UK; 7 Blizard Institute, Barts and the London School of Medicine, Queen Mary University of London, London, UK; 8 Lee Kong Chian School of Medicine, Nanyang Technological University, Singapore, Singapore; 9 Department of Psychiatry and Neurochemistry, Institute of Neuroscience and Physiology, The Sahlgrenska Academy at the University of Gothenburg, Gothenburg, Sweden; 10 UK Dementia Research Institute at UCL, London, UK

**Keywords:** Down syndrome, Alzheimer’s disease, Dementia, Neurofilament light, Biomarker

## Abstract

**Background:**

Down syndrome (DS) may be considered a genetic form of Alzheimer’s disease (AD) due to universal development of AD neuropathology, but diagnosis and treatment trials are hampered by a lack of reliable blood biomarkers. A potential biomarker is neurofilament light (NF-L), due to its association with axonal damage in neurodegenerative conditions.

**Methods:**

We measured blood NF-L concentrations in 100 adults with DS using Simoa NF-light® assays, and we examined relationships with age as well as cross-sectional and longitudinal dementia diagnosis.

**Results:**

NF-L concentrations increased with age (Spearman’s rho = 0.789, *p* < 0.001), with a steep increase after age 40, and they were predictive of dementia status (*p* = 0.022 adjusting for age, sex, and *APOE4*), but they showed no relationship with long-standing epilepsy or premorbid ability. Baseline NF-L concentrations were associated with longitudinal dementia status.

**Conclusions:**

NF-L is a biomarker for neurodegeneration in DS with potential for use in future clinical trials to prevent or delay dementia.

## Background

Down syndrome (DS), caused by the trisomy, translocation, or partial trisomy of chromosome 21, is the most common genetic cause of intellectual disability (ID), with an estimated population prevalence of 6 million worldwide. Dementia is a common feature of the aging process in DS that is due to the triplication of the amyloid precursor protein on chromosome 21, leading to brain pathology indicative of Alzheimer’s disease (AD) [1], with a cumulative incidence for dementia in excess of 90% by the age of 65 [2] and a mean age at dementia diagnosis of 55 [3]. DS is therefore a genetic form of AD alongside autosomal dominant causes of AD [4].

Neurofilament light (NF-L) is one of the scaffolding cytoskeleton proteins of myelinated subcortical axons [5] and can now be reliably measured in blood using ultrasensitive single-molecule array (Simoa) technology. Blood concentration of NF-L correlates well with corresponding cerebrospinal fluid (CSF) measures [6] and reflects axonal damage in neurological disorders, including frontotemporal dementia [7], multiple sclerosis [8], and familial and sporadic AD [9, 10]. NF-L correlates with other measures of disease stage and severity [10], but the utility of NF-L in populations with other genetic forms of AD is yet to be fully explored.

Aging in DS is invariably associated with AD pathology, and this condition therefore presents an opportunity to confirm the relationship between NF-L and progression of AD pathology. Furthermore, DS is a critically important group for clinical trials of treatments to prevent and delay AD pathology and dementia symptoms, and a reliable blood biomarker would help to address disease burden in this vulnerable population by enabling clinical trials of disease-modifying treatments. In the present study, we aimed to explore the relationship between plasma NF-L levels, age, and dementia status in individuals with DS, as well as its independence from sex effects, premorbid intellectual ability levels, and long-standing epilepsy.

## Methods

Participants aged 16 years and older were recruited across England via care homes, support groups, and local NHS sites. Participants with an acute physical or mental health condition were excluded until they had recovered; other details of the cohort have been described previously [11]. DS status was confirmed using DNA from saliva or blood and genotyped using OmniExpressExome arrays (Illumina, San Diego, CA, USA); trisomy status was visually confirmed in GenomeStudio software (Illumina) (*see* Table 1). Apolipoprotein E (*APOE*) status was determined using TaqMan assays for rs7412 and rs429358 (Thermo Fisher Scientific, Waltham, MA, USA).

**Table 1:** Demographics of all participants included in group and subgroup analyses

	All participants	Dementia (baseline)	No dementia (baseline)	Participants with follow-up data
Number of subjects	94	18	76	29
Age at baseline, years, mean ± SD (range)	42.68 ± 14.87 (17–73)	55.17 ± 9.92 (40–69)	39.72 ± 14.34 (17–73)	52.63 ± 8.88 (40–72)
DS type	89 (94.7%) trisomy, 2 (2.1%) translocation, 3 (3.2%) unknown	18 (100.0%) trisomy	71 (93.4%) trisomy, 2 (2.6%) translocation, 3 (3.9%) unknown	28 (96.6%) trisomy, 1 (3.4%) unknown
Sex	41 (43.6%) female, 53 (56.4%) male	6 (33.3%) female, 12 (66.7%) male	35 (46.1%) female, 41 (55.9%) male	10 (34.5%) female, 19 (65.5%) male
Ethnicity	85 (90.4%) white, 9 (9.6%) other	17 (94.4.0%) white, 1 (5.6%) other	68 (89.5%) white, 8 (10.5%) other	27 (93.1%) white, 2 (6.8%) other
Predementia ID level	37 (39.4%) mild, 47 (50%) moderate, 9 (9.6%) severe, 1 (1.2%) unknown	6 (33.3%) mild, 9 (50.0%) moderate, 3 (16.7%) severe	31 (40.8%) mild, 38 (50%) moderate, 6 (7.9%) severe, 1 (1.3%) unknown	13 (44.8%) mild, 12 (41.4%) moderate, 3 (10.3%) severe, 1 (3.4%) unknown
APOE status	68 (72.3%) non-*APOE4* carrier, 23 (24.5%) *APOE4* carrier, 3 (3.2%) unknown	12 (66.7%) non-*APOE4* carrier, 5 (27.8%) *APOE4* carrier, 1 (5.5%) unknown	56 (73.7%) non-*APOE4* carrier, 18 (23.7%) *APOE4* carrier, 2 (2.6%) unknown	22 (75.9%) non-*APOE4 *carriers, 6 (20.7%) *APOE4 *carrier, 1 (3.4%) unknown
NF-L level, ng/L, median (range)	22.74 (6.11–136.91)	63.76 (15.21–136.91)	19.96 (6.11–116.84)	32.67 (12.23–481.97)

*Abbreviations: DS* Down syndrome, *ID* Intellectual disability, *APOE* Apolipoprotein E, *APOE4*, Apolipoprotein E E4 allele, *NF-L* Neurofilament light

Assessment included a detailed interview with carers using the Cambridge Examination of Mental Disorders of Older People with Down’s syndrome and others with intellectual disabilities [12] to determine decline in several domains, including memory. Premorbid ID level was defined according to the International Classification of Diseases, Tenth Revision, diagnostic system’s descriptions of mild, moderate, and severe ID, based on carers’ reports of the individuals’ best-ever level of functioning [13]. At baseline, dementia was defined as a confirmed clinical diagnosis. At follow-up, participants were classified according to whether they had retained or been given a diagnosis of dementia or were being investigated for dementia.

Blood samples from 100 individuals were collected in lithium heparin tubes (Fisher Scientific UK, Loughborough, UK) and sent overnight for processing. Blood was layered over a similar amount of Ficoll (GE Healthcare, Little Chalfont, UK), then centrifuged in a swing-out rotor for 40 minutes at 400 × *g* without braking. Plasma samples were stored at − 80 **°**C. Plasma NF-L concentration was measured by the same laboratory technician with reagents from a single lot using the Simoa NF-light® assay (a digital sandwich immunoassay employing antibodies directed against the rod domain of NF-L) on an HD-1 Simoa analyzer according to the protocol issued by the manufacturer (Quanterix, Lexington, MA, USA). Samples were run in duplicate, and coefficients of variation (CVs) for duplicates were set to be < 12%. All samples measured within the range spanned by the limits of quantification and interassay CV for the high- and low-concentration quality controls were 6.6% and 8.1%, respectively.

All statistical tests were two-sided, and statistical significance was set at *p* < 0.05. We tested associations between plasma NF-L samples and demographic or clinical factors using Mann-Whiney *U*, Kruskal-Wallis, and Spearman’s rank correlation tests as appropriate. Associations between plasma NF-L and dementia diagnosis were tested using logistic regression and log-transformed NF-L values, with adjustment for age and sex; we also adjusted for *APOE4 *status cross-sectionally.

## Results

NF-L levels were obtained from 100 participants (age range 17–73 years). Five results were excluded after failing to meet CV thresholds, meaning 95 adults were included in subsequent analyses. Of adults aged 36 and older who are being targeted for longitudinal follow-up, 29 of 63 (46%) had completed a follow-up assessment at the time of this report (mean number of months between assessments 23.4, SD 3.9). One individual had experienced an occlusive cerebrovascular event 4–6 months prior to donating the blood sample and converted to dementia status at follow-up but was an outlier with an NF-L level of 481.97 ng/L; thus, this individual was excluded from cross-sectional analysis. Among the remaining 94 individuals, NF-L concentration had a median value of 22.74 ng/L (range 6.11–136.91 ng/L). At baseline, 18 of 94 participants had a clinical diagnosis of dementia (Table 1).

### NF-L levels and relationship with dementia and other clinical variables

**Fig. 1:**
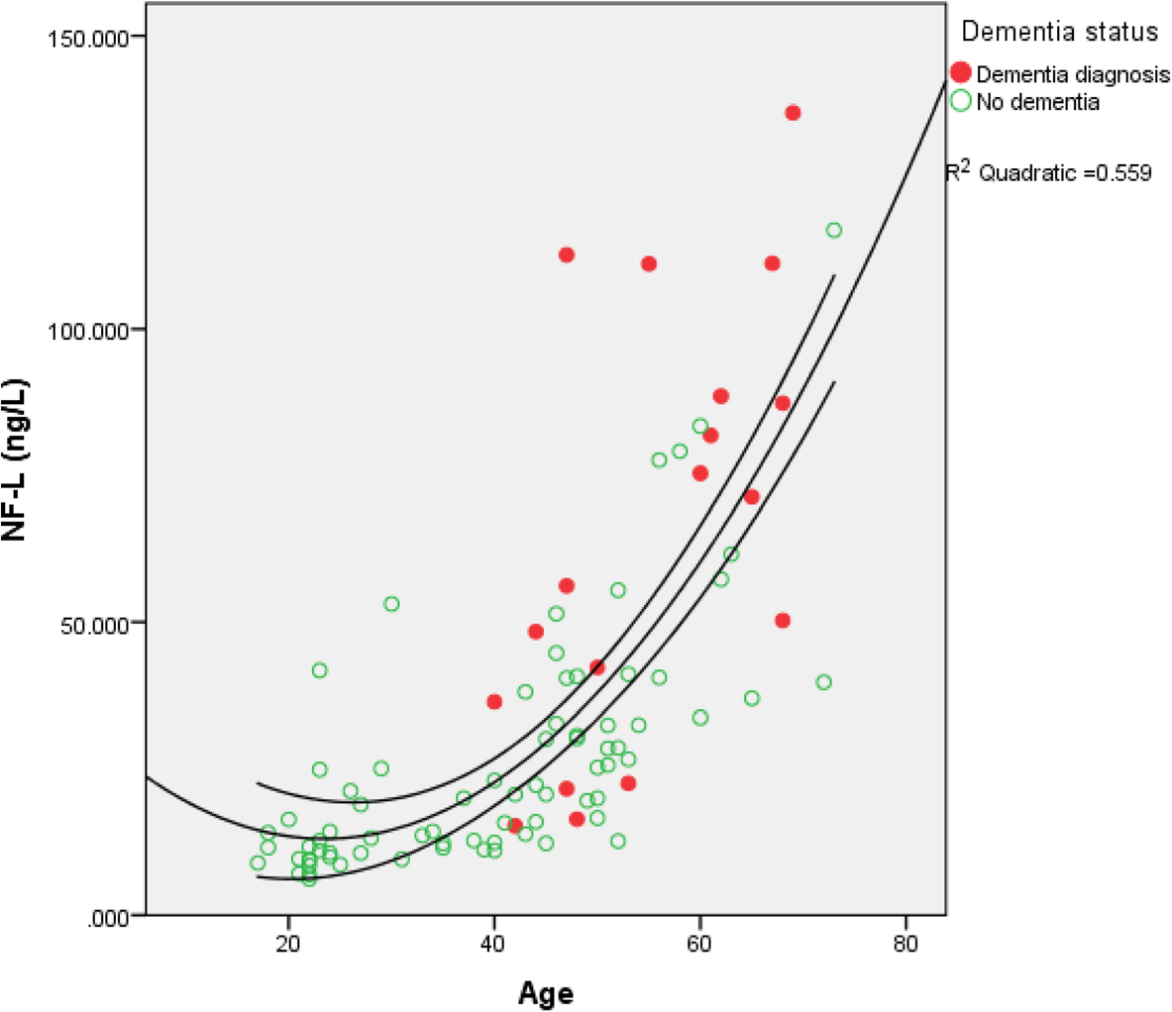
Neurofilament light (NF-L) concentration by age and dementia status of individuals with Down syndrome

NF-L levels did not differ by premorbid ID level (Kruskal-Wallis test, *p* = 0.195), sex (Mann-Whitney *U* test, *p* = 0.837) or long-standing epilepsy (Mann-Whitney *U* test, *p* = 0.858). NF-L level and age of participants were significantly correlated (Spearman’s rho = 0.789, *p* < 0.001) (Fig. 1), such that those aged 35 and older had significantly higher levels of NF-L than younger individuals (median 11.52 ng/L vs. 32.42 ng/L, Mann-Whitney *U* test *p* < 0.001).

Those with dementia had significantly higher levels of NF-L (median 63.76 ng/L vs. 19.96 ng/L; Mann-Whitney *U* test *p* < 0.001), and a logistic regression model adjusting for age, sex, and APOE4 status revealed that NF-L levels remained predictive of dementia status (*p* = 0.022).

### NF-L and predictive validity for dementia

Seven (24.1%) of 29 individuals with follow-up cognitive data had a clinical diagnosis of dementia at baseline, all with the dementia diagnosis retained at follow-up, with a further 2 (6.9%) individuals converting to dementia status by follow-up, whereas 3 (10.3%) participants were under investigation for dementia at follow-up. Predictive validity of NF-L levels at baseline was explored by combining individuals with confirmed or suspected dementia at follow-up (*n* = 12, median NF-L 77.38 ng/L) and comparing them with those who remained dementia-free (median NF-L 19.94 ng/L). Higher levels of NF-L at baseline predicted the likelihood of dementia at follow-up, even when adjusted for age and sex (*p* = 0.036).

## Discussion

We have demonstrated that NF-L measured in blood using an ultrasensitive assay is strongly associated with age and dementia status in individuals with DS, and baseline levels were predictive of dementia diagnosis over time. Furthermore, NF-L levels did not differ according to severity of premorbid ID or by long-standing epilepsy diagnosis (a common neurological comorbidity in DS), suggesting that it is a stable and feasible biomarker that can be used in clinical populations.

Our results indicate that this marker could pinpoint the onset of neurodegeneration in DS. NF-L showed an age relationship in keeping with postmortem data and amyloid positron emission tomography studies of AD pathology in adults with DS [1, 14, 15]. In familial AD, serum NF-L concentration is increased prior to symptom onset and correlates with measures of disease stage and severity [10]. In sporadic AD, plasma NF-L concentration is increased already in the mild cognitive impairment stage of the disease and correlates with cognitive hallmarks of the disease [9]. Our results show that NF-L has similar relationships with clinical dementia diagnoses in DS. Substantial neurodegeneration associated with the buildup of amyloid likely occurs before a threshold for dementia diagnosis is reached, and like the presence of amyloid plaques preceding the clinical signs by several decades, NF-L levels may be increased some time before onset of significant symptoms, in keeping with some degree of neurodegeneration prior to clinical impact. Further longitudinal studies may elucidate the relationship between NF-L levels and development of symptoms in genetically predisposed populations such as DS.

Although NF-L is a marker of axonal damage, and thus not specific to AD [7–9, 16], in a population such as DS where AD is almost always the cause of dementia, the lack of specificity is arguably less of an issue, and NF-L could potentially be used as a biomarker in treatment trials. Recent studies in rodent models of neurodegenerative diseases showed that NF-L levels in CSF and plasma responded to experimental manipulation and targeted therapy [17], and normalization of serum/plasma NF-L in response to treatment has already been demonstrated in patients with multiple sclerosis [8, 18]. These findings suggest that NF-L may be a useful biomarker of response to treatment.

The relationship between serum NF-L levels and genetic markers and other AD biomarkers, including markers of inflammation and oxidative stress (both of which are increased in DS and associated with cognitive decline [19–22]), could also be explored in future research to refine prediction of cognitive decline and to stratify patients with Down syndrome according to dementia risk for clinical trials of potential treatments.

## Conclusions

Although further work is required to establish long-term predictive and concurrent validity of NF-L, our data suggest that this biomarker could be instrumental in allowing an experimental medicine approach in individuals with DS and other high-risk populations to test treatments that might prevent or delay dementia onset.
